# Supplementary material for: Phytochemical Study on Seeds of Paeonia clusii subsp. rhodia—Antioxidant and Anti-Tyrosinase Properties
Source: Int J Mol Sci. 2023 Mar 3;24(5):4935. doi: 10.3390/ijms24054935 (PMC10003135; doi:10.3390/ijms24054935)
Supplement: Supplementary file 1 [file ijms-24-04935-s001.zip › ijms-2153277-supplementary.pdf]

International Journal of *Molecular Sciences*

MDPI

SUPPLEMENTARY FILE

Phytochemical study on seeds of *Paeonia clusii* subsp. *rhodia*. Antioxidant and Anti-tyrosinase properties

Vithleem Klontza, Konstantia Graikou, Antigoni Cheilari, Vasilios Kasapis, Christos Ganos, Nektarios Aligiannis, Ioanna Chinou*

Lab. of Pharmacognosy and Chemistry of Natural Products, Faculty of Pharmacy, National and Kapodistrian University of Athens, Panepistimiopolis, Zografou, 15771, Greece. maklobetty@gmail.com; kgraikou@pharm.uoa.gr; cheilarianti@pharm.uoa.gr; kasapis.b@gmail.com; chris50ganos.hotmail.com; aligiannis@pharm.uoa.gr

*Correspondence: ichinou@pharm.uoa.gr;

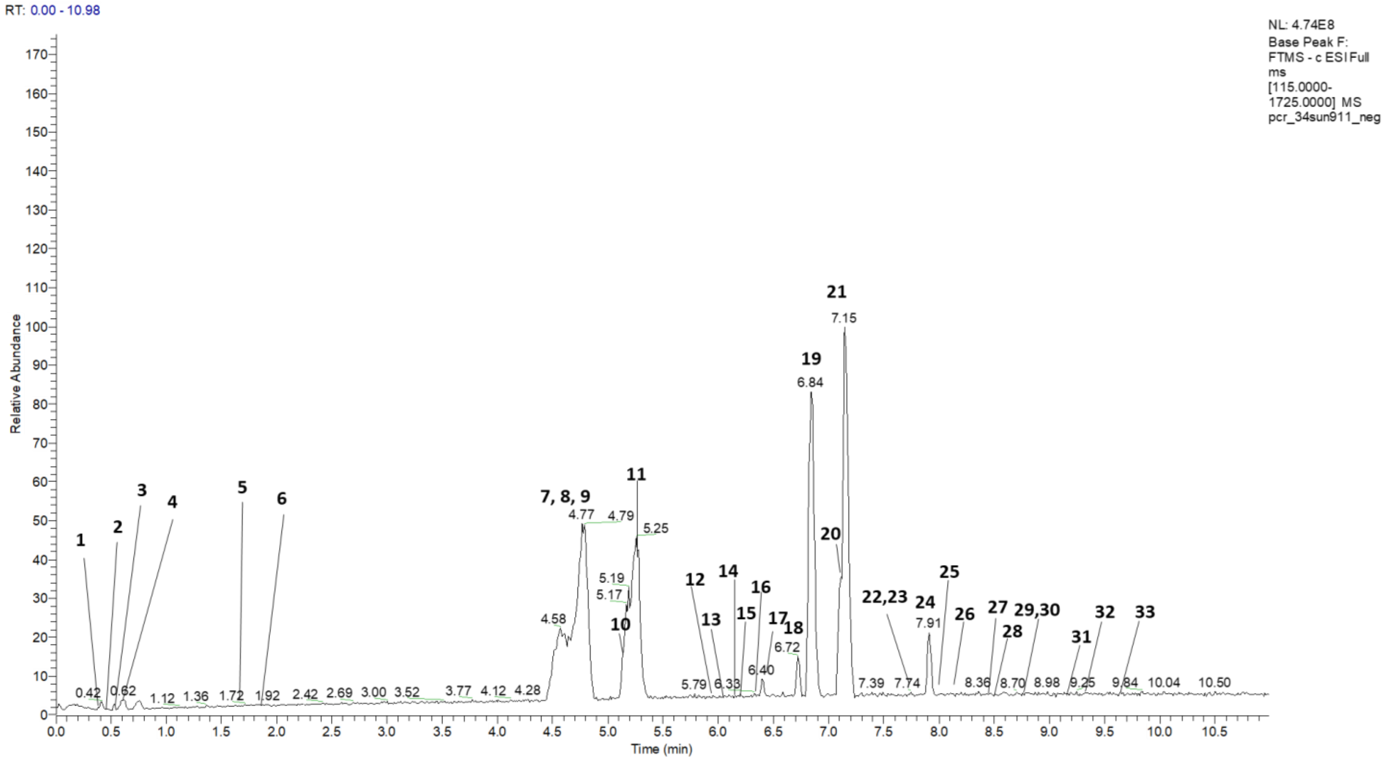

Figure S1. LC-MS chromatogram of the black seeds of *P. clusii subsp. rhodia* by UHPLC-HRMS analysis
